# Lifestyle modification intervention among pregnant women with hypertension based on the self-determination theory using M-Health

**DOI:** 10.3389/fpubh.2025.1495281

**Published:** 2025-06-02

**Authors:** Negar Heidari, Fatemeh Rajati, Paria Heidari, Mojgan Rajati

**Affiliations:** ^1^Department of Health Promotion and Education, School of Health, Kermanshah University of Medical Sciences, Kermanshah, Iran; ^2^Department of Obstetrics and Gynecology, School of Medicine, Kermanshah University of Medical Sciences, Kermanshah, Iran

**Keywords:** mobile application, hypertension, lifestyle, pregnant women, self-determination theory, M-health

## Abstract

**Background:**

Digital health technologies have the potential to empower patients and enhance the management of chronic diseases, such as hypertension, which often suffers from low awareness and control rates in developing countries. This study aims to investigate the effectiveness of a lifestyle intervention tailored for hypertensive pregnant women, employing strategies derived from self-determination theory through a mobile health program.

**Methods:**

The study utilized an experimental pre-test/post-test design with a two-month follow-up period. It examined the impact of an educational intervention on pregnant women with hypertension in Kermanshah, Iran, from 2021 to 2023. Sixty pregnant women with hypertensive disorders were recruited from medical centers and randomly assigned to either the intervention group (*N* = 30) or the control group (*N* = 30). A mobile app was employed to deliver the educational intervention, addressing lifestyle factors such as nutrition, physical activity, and stress management. The app’s effectiveness was assessed based on multiple criteria, including content quality, data accuracy, decision support, language and cultural sensitivity, user feedback interpretation, and personalized recommendations.

**Results:**

After the intervention, repeated measures ANOVA indicated that the intervention resulted in statistically significant improvements in all study variables within the intervention group compared to the control group (*p* < 0.05), with the exception of blood pressure (*p* > 0.05). These changes remained significant during the follow-up period, except for relatedness, moderate physical activity, sitting time, and total physical activity (*p* > 0.05). The intervention significantly enhanced both controlled autonomy (*p* < 0.001, η^2^ = 0.21) and autonomous autonomy (*p* < 0.001, η^2^ = 0.30), as well as competence (*p* < 0.001, η^2^ = 0.27). The effect on relatedness was marginally non-significant (*p* = 0.053, η^2^ = 0.053). Improvements in nutrition, physical activity, and perceived stress were significant across between-group, within-group, and interaction effects (*p* < 0.05). All levels of physical activity showed significant improvements (*p* < 0.05), except for low physical activity (*p* > 0.05). There were significant between-group differences in both systolic and diastolic blood pressure (*p* < 0.05), but no significant within-group or interaction effects were observed.

**Conclusion:**

The M-Health intervention led to improvements in lifestyle factors and self-determination constructs, with the exception of relatedness, which may be attributed to the limited features of the app. Although blood pressure did not change significantly, the reduction in systolic pressure could still be clinically meaningful. M-Health interventions grounded in self-determination theory show promise for supporting hypertensive pregnant women.

## Introduction

Hypertensive disorders during pregnancy contribute significantly to maternal mortality and affect 5–10% of pregnant women, impacting maternal, fetal, and neonatal health (1, 2). In Iran, nearly half of these cases remain uncontrolled (3), with a study in Ilam showing 3.54% of pregnant women experiencing hypertension (4).

Hypertension in pregnancy, as defined by the International Society for the Study of Hypertension in Pregnancy (ISSHP), is characterized by a systolic blood pressure (sBP) of ≥140 mm Hg and/or a diastolic blood pressure (dBP) of ≥90 mm Hg. The ISSHP classifies hypertensive disorders of pregnancy based on gestational age, identifying chronic, white coat, and masked hypertension before 20 weeks, while after 20 weeks, it defines gestational hypertension and pre-eclampsia based on the presence of new-onset hypertension, proteinuria, and organ dysfunction (5). Lifestyle modifications, including weight management, promoting physical activity, and reducing stress, are recommended for pregnant women with hypertension (6).

The rapid advancement of digital health technologies presents transformative opportunities for managing chronic diseases (7). Telemedicine, e-health, and M-Health solutions empower patients to actively monitor and improve their health while enhancing health care (8). These innovative approaches are particularly important for managing chronic conditions such as hypertension, which require ongoing self-care and regular interactions between patients and their healthcare teams (9). As pregnant women may face some infrastructural challenges in accessing healthcare centers, M-Health apps and wearable devices can eliminate the need for physical referrals. They provide hypertensive pregnant women with real-time feedback, personalized education, and self-monitoring tools to encourage the adoption of healthier behaviors (10)

Research indicates that a sense of internal independence and competence, along with positive relationships in family and social environments, is crucial for adopting healthy lifestyles and improving health-related behaviors (11, 12). These factors are highlighted by self-determination theory, Self-determination theory emphasizes that fulfilling the innate human needs for autonomy, competence, and relatedness enhances intrinsic motivation, leading to better well-being and personal growth (13, 14).

Smartphone technology has become widely adopted globally, with mobile phone penetration rates exceeding 85% worldwide (15). In Iran, this rate increased dramatically from 22% in 2006 to 104.13% by 2017 (16), positioning the country 17th globally with a penetration rate of 43.20% (17). The rise of smartphones has opened new opportunities to improve access to healthcare services and support self-management of health conditions through mHealth (18), with numerous studies reporting generally positive outcomes for mobile applications (19–21).

M-Health apps and wearable devices offer hypertensive pregnant women real-time feedback, personalized education, and self-monitoring tools, such as diet tracking and medication reminders, empowering them to manage their condition actively and communicate effectively with healthcare providers (10).

There is a significant gap in understanding how lifestyle interventions for pregnant women can align with educational theories that address their specific needs. While the self-determination theory is recognized as important for M-Health interventions, its application in comprehensive lifestyle programs for pregnant women remains underexplored, indicating a need for research. Integrating M-Health into comprehensive lifestyle modification programs based on self-determination theory may help address the unique psychological and social needs of this population, ultimately leading to improved hypertension control and better maternal-fetal outcomes. This study aims to explore the effectiveness of a lifestyle intervention designed for hypertensive pregnant women, employing strategies from self-determination theory in the form of an M-Health program.

## Materials and methods

### Study design and participants

The research employed an experimental approach and utilized a pre-test/post-test design with a two-month follow-up period to investigate the impact of an intervention on pregnant women with hypertension in Kermanshah from 2021 to 2023. After coordinating with the head of the health center, 60 pregnant women with hypertension who attended the medical centers of Kermanshah University of Medical Sciences were included in the study through convenience sampling.

The participants in the intervention group were informed about the importance of not preeclampsia, and chronic hypertension., literacy, access to a mobile phone and internet, while exclusion criteria included, visual or auditory impairments, and underlying conditions like gestational diabetes, arthritis, cancer, and autoimmune diseases. Participants had to be at least 3 months away from their expected delivery date and in a stable condition. Pregnant women with 3 months remaining until birth were included in the study to ensure they completed at least the 1-month intervention and 2 months follow-up assessment before delivery, considering the physiological changes after delivery.

Pregnant women with hypertensive disorders were selected based on their active health records, which had been previously diagnosed at healthcare centers. Additionally, for recruitment, their blood pressure measurements were obtained and verified by a physician in accordance with the Joint National Committee (JNC-7) guidelines (22).

### Procedure

The participants were then randomly assigned to either the intervention or control group, with 30 individuals in each group, using random allocation software^1^. After explaining the aim of the study, all participants signed a written informed consent form. The participants in the intervention group were informed about the importance of not sharing information with those in the control group. Periodic checks were conducted with the participants to prevent any contamination of information between the intervention and control groups, ensuring that there was no interaction or exchange of information between the two groups.

The educational intervention was conducted using the M-Health application “Pregnant Women,” which was designed by the researchers. The content of the application covered various topics related to lifestyle and blood pressure, including Dietary Approaches to Stop Hypertension (DASH) (23), physical activity (24), and stress management. For 6 weeks, in line with similar studies, this content was provided to the pregnant women in the intervention group (25, 26). The control group did not receive any intervention during the study and only received routine care to manage their hypertension, in accordance with the National Guide for Providing Midwifery and Delivery Services (27). However, following the completion of the study, the control group was provided with the same intervention for ethical reasons.

The nutrition content includes the main food groups and their recommended consumption, harmful foods during pregnancy, and information about fast food consumption (including the risks associated with fast food for both the mother and fetus, methods for controlling fast food intake, and healthier alternatives). It also covers salt consumption (such as the recommended amount of salt during pregnancy, the dangers of excessive salt intake, sources of salt, strategies for reducing salt consumption, and the risks of consuming sea salt during pregnancy) and the use of spices (identifying harmful and non-harmful spices during pregnancy) (28). Pregnant women were advised to engage in walking or light to moderate physical activity at least three times a week.

Pregnant women were advised to avoid certain exercises that could be risky for both them and their baby, including jumping, back-arching positions, and heavy weightlifting. They were also instructed to watch for warning signs during exercise, such as uterine contractions, vaginal bleeding, and severe shortness of breath. If they experience any of these symptoms, it’s important to stop exercising right away and contact a doctor (29).

The healthy lifestyle program for pregnant women included a variety of relaxation techniques aimed at alleviating stress. Applied relaxation methods, such as muscle relaxation and general relaxation techniques, were incorporated due to their significant impact on reducing stress and promoting empowerment. The program also educated women about the benefits of acute relaxation methods, such as listening to music or engaging in guided imagery, which can help reduce physiological indicators of stress. Additionally, aromatherapy was included as a complementary therapy within the program.

This study utilized strategies from self-determination theory to support pregnant women’s needs for autonomy, competence, and relatedness, thereby enhancing their motivation for lifestyle changes to manage blood pressure. A goal-setting feature was integrated into the M-Health application to promote autonomy, allowing women to define their own personal goals for various activities. This approach helps them feel more in control by emphasizing their internal motivation and understanding their ability to achieve health objectives.

To address the need for competence, feedback, history, and self-monitoring strategies were employed. The feedback feature provided users with comprehensive details about their progress in various activities, accompanied by encouraging messages. Additionally, it presented cumulative statistics over specified time periods (e.g., a week or a month), helping users assess trends over time and compare their current performance with past performance. Positive feedback resulting from behavior performance, as provided by a gynecologist, highlighted growth or improvement trends, which can enhance an individual’s sense of competence.

The history feature offered a representation of a person’s activities over time. By observing what occurred on different days, individuals could relate their successes or failures in achieving goals to contextual factors that may influence their performance. This information helps individuals gain self-awareness and understand how their personal circumstances may affect their behavior, thereby supporting their basic need for competence.

Furthermore, the self-monitoring feature enabled users to document their goal achievements or task completions related to specific activities. Each recorded activity served as confirmation of its completion, further bolstering the user’s sense of competence.

To address the need for relatedness, we employed the following strategies: (a) Peer Comparison: This functionality typically includes a list of users engaged in the same activity, sorted by goal achievement and featuring users’ usernames. Through peer comparison, users can evaluate their performance relative to others, potentially enhancing their sense of effectiveness. As a result, this feature supports interpersonal communication and contributes to the basic need for competence by allowing individuals to assess their efficiency and mastery of specific activities. (b) Challenging Peers: This feature allows users to challenge others to achieve a specific goal, which can be targeted at an individual or a group. Challenges can be private (involving friends and family) or public (involving random users). Competing with others enables users to compare their performance outcomes, fostering interaction and mutual observation. This feature enhances communication by facilitating interpersonal connections, recognition, and trust among individuals. (c) Messaging Feature: This feature enables users to communicate with a doctor by sending text messages and receiving responses. When individuals feel that their need for communication is met, they are more likely to be intrinsically motivated and able to sustain their activities over time.

The intervention group received the M-Health application for a duration of 6 weeks, following an educational design. Demographic variables, basic psychological needs based on Self-Determination Theory (autonomy, competence, and relatedness), and healthy lifestyle variables, including nutrition, physical activity, and stress management, were assessed before the intervention, immediately after, and 2 months post-intervention of the M-Health intervention.

### M-Health application

We used the following criteria to assess the accuracy of the M-health application: (a) Content Accuracy: The information provided in the application regarding pregnancy, complications, maternal health, and self-care was verified to ensure it aligns with current medical guidelines and best practices in Iran. (b) Data Accuracy: User-inputted data, such as health metrics (e.g., blood pressure, weight, fetal movements), was accurately recorded and processed by the application. (c) Decision Support Accuracy: When the application offered decision support or guidance for specific situations (e.g., when to seek medical attention), we ensured that the recommendations were consistent with professional medical advice and standards. (d) Language and Cultural Sensitivity: We ensured that diverse cultural backgrounds were considered, and that the content and features of the application were presented in a language that resonates with hypertensive pregnant women. (e) User Feedback Interpretation: We assessed how the application processed and interpreted feedback or input from hypertensive women to provide personalized recommendations or support, ensuring that the needs and preferences of the women were accurately reflected. (f) Personalized Recommendations: The accuracy of any personalized recommendations or interventions provided by the application was evaluated based on a pilot study involving 10 hypertensive pregnant women, taking into account their input and health data.

### Measurements

This study utilized a comprehensive set of measurement instruments as follows:

#### Demographic information

This questionnaire collects basic data about participants, which may include age, gestational age, weight before pregnancy, BMI, number of gravida, number of the abortion, taking antihypertensive drugs, education level, and employment status.

#### Health behavior (dietary sodium)

This section assesses participants’ dietary sodium intake, focusing on health behaviors related to nutrition. It aims to understand how dietary choices affect overall health.

#### International physical activity questionnaire (IPAQ)

The IPAQ was employed to evaluate physical activity across various domains, including leisure-time, domestic, work, and transport-related activities. Each domain measures walking, moderate, and vigorous physical activities performed for at least 10 consecutive minutes daily over 7 days. The average metabolic equivalent (MET) score for total weekly physical activity is calculated using the formula:


$$\begin{array}{l} \text{Total physical activity in}\;\text{MET}-\text{minutes}/\text{week}\\ =\text{sum}\;\text{of}\;\text{MET}\;\text{minutes for Walking}+\text{Moderate}\\ +\text{Vigorous activities} \end{array}$$

Individual MET scores for each activity type are computed within their domains and combined to produce a total score, using the equation:


$$\begin{array}{l} \text{Total}\;\text{MET}-\text{minutes}/\text{week}=\text{Met}-\text{level}\\ \times \;\text{minutes}\;\text{per}\;\text{day}\times \text{days}\;\text{per}\;\text{week}, \end{array}$$

where 1 MET represents resting energy expenditure.

Sitting behavior was also measured by hour per day (30).

#### Perceived stress questionnaire

The Perceived Stress Scale (PSS), developed by Cohen et al. in 1983, consists of 14 items scored from 0 to 4, with a total score range of 0 to 56. The internal consistency ranges from Cronbach’s alpha of 0.84 to 0.80 (31).

#### Treatment self-regulation questionnaire

The measures comprise three subscales of autonomous regulation (six items), controlled regulation (six items), and amotivation (three items). We report only the two first subscales for the measurement of autonomous in this study.

- *Autonomous Regulation Subscale:* Measures intrinsic motivation and self-determined regulation, reflecting the extent to which individuals engage in behaviors because they find them meaningful and enjoyable.
- *Controlled Regulation Subscale:* Evaluates extrinsic motivation and controlled regulation, indicating how much behavior is driven by external pressures or obligations.

Scores are calculated based on specific questions related to each subscale, with a scoring range from 1 (not correct) to 7 (completely correct). Validity and reliability of the questionnaire in persion population were approved by Cronbach’s alpha = 0.893 and ICC = 0.982 (32). We assessed the content validity index (CVI = 0.95) and content validity ratio (CVR = 0.8) in this study.

#### Perceived Competence Scale

The Perceived Competence Scale for Self-Management of Diabetes (PCSD) is a four-item tool that evaluates patients’ self-perceived daily disease management abilities on a seven-point scale, with total scores ranging from 4 (indicating low competence) to 28 (indicating high competence) (33). We evaluated the measures of content validity index (CVI = 0.97) and content validity ratio (CVR = 0.84) in the current study. In this study, we analyzed the content validity index (CVI = 0.79) and the content validity ratio (CVR = 0.98). This scale was used to measure competency.

#### Health care climate questionnaire

This questionnaire evaluates the supportiveness of healthcare providers regarding patient independence. It consists of six questions, with higher average scores indicating a greater perception of support from healthcare professionals. Responses are measured on a 7-point Likert scale (from completely disagree: 1 to completely agree: 7). The reliability index of Cronbach’s alpha was *α* = 0.945, with ICC = 0.979. The CVI values for all the items were equal to or above 0.8 and the CVR value for the total scale was 0.91 in Iranian version (34). This scale was used to evaluate relatedness.

#### Blood pressure measurements

Women’s blood pressure was recorded at three different time points: before the intervention, immediately after, and 2 months following the intervention. This helps assess the impact of the intervention on systolic and diastolic blood pressure over time.

### Statistical analysis

The distribution of demographic variables in the two intervention and control groups was compared using the independent *t*-test and chi-square test.

To compare the scores of autonomy, competence, relatedness, as well as behaviors related to nutrition, physical activity, and stress, along with systolic and diastolic blood pressure measurements before the intervention, immediately after, and 2 months post-intervention in the two study groups, repeated measures analysis of variance (ANOVA) was conducted. The assumptions of homogeneity of variances were made by the Machley sphericity test. Stated violations of sphericity were corrected with Greenhouse–Geisser correction (35). Partial Eta-squared for assessing effect sizes was considered for mixed ANOVA with repeated measures. The effect of the group The significance level for interpreting the findings was set at 0.05. The collected data were entered into SPSS software version 27, and descriptive statistics methods (mean and standard deviation) were applied.

### Findings

The results of the independent sample *t*-test showed that the mean age of the pregnant women in the intervention and control groups was 30.03 ± 3.27 and 29.26 ± 2.71 years, respectively (*p*-value = 0.328). The average gestational age in both the intervention and control groups was 21.16 ± 2.30 and 20.20 ± 2.17 weeks, respectively (*p*-value = 0.954). The BMI of pregnant women before pregnancy in the two intervention and control groups was calculated as 25.16 ± 1.97 and 25.02 ± 1.74, respectively (*p*-value = 0.766) (Table 1).

**Table 1:** Descriptive analysis of demographic variables.

Variable	Group	Control	Intervention	*p*-value
Wemens age (Mean ± SD)		29.26 ± 2.71	30.03 ± 3.27	0.328*
Gestational age (Mean ± SD)		20.20 ± 2.17	21.16 ± 2.30	0.954*
Weight before pregnancy (Mean ± SD)		64.06 ± 4.77	63.66 ± 5.49	0.765*
Height (Mean ± SD)		160.10 ± 6	159.06 ± 4.59	0.457*
BMI (Mean ± SD)		25.02 ± 1.74	25.16 ± 1.97	0.766*
Gravida *n* (%)	1	9 (30)	11 (36.7)	0.698**
	2	15 (50)	13 (43.3)	
	3	6 (20)	5 (16.7)	
	4	0 (0)	1 (3.3)	
Number of abortions *n* (%)	No abortion	25 (83.3)	23 (76.7)	0.519**
	Having abortion	5 (16.7)	7 (23.3)	
Taking antihypertensive drugs *n* (%)	No use	22 (73.3)	27 (90)	0.688**
	Use	8 (26.7)	3 (10)	
Level of education *n* (%)	Secondary Education	3 (10)	5 (16.7)	0.547**
	Diploma	21 (70)	17 (56.7)	
	Academic Education	6 (20)	8 (26.7)	
Employment status *n* (%)	Employed	5 (16.7)	8 (26.7)	0.321**
	Housewife	24 (80)	19 (63.3)	
	Working at home	1 (3.3)	3 (10)	

* Independent sample *t*-test. ** Chi-square test.

The results of the Chi-square test indicated that there was no significant difference in the proportions of gravida, the number of abortions, the use of antihypertensive drugs, education, and employment status between the intervention and control groups at baseline. From all, 43.3% of pregnant women in the intervention group and 50% in the control group had experienced two pregnancies (*p*-value = 0.698). The majority of women in both the intervention (76.7%) and control (83.3%) groups reported no history of abortions (*p*-value = 0.519). Furthermore, a significant proportion of women in the intervention (90%) and control (73.3%) groups did not report the use of antihypertensive drugs (*p*-value = 0.688). In terms of education, most pregnant women in the intervention group (56.7%) and the control group (70%) had completed high school. Additionally, the majority of pregnant women in the intervention (63.3%) and control (80%) groups were identified as housewives (Table 1).

According to Table 2, the result of independent sample *t*-test shows all study variables before the intervention exhibited no statistically significant differences between the intervention and control groups (*p-*value >0.05). The implementation of the M-Health for intervention resulted in a statistically significant increase in all study variables (*p-*value <0.05), except for the blood pressure variables, in the intervention group compared to the control group (*p-*value >0.05). These changes remained statistically significant during the follow-up period (*p-*value <0.05), except for ralatedness (*p*-value = 0.20), moderate physical activity (*p*-value = 0.93), sitting (*p*-value = 0.09), and total score of physical activity (*p*-value = 0.10).

**Table 2:** The effect of time, group, and interaction effect of time and group on outcome variables before, after, and 2-month follow up.

Variables	Time	Group	Before	After	2-month follow up	Group			Time			group × Time		
						η^2^	*p*-value	*F*	η^2^	*p*-value	*F*	η^2^	*p*-value	*F*
Controlled autonomy	Intervention		32.86 ± 3.53	25.33 ± 5.04	27.13 ± 3.83	40.7	< 0.001	0.412	10.36	0.002	0.152	15.7	< 0.001	0.213
	Control		31.90 ± 4.03	30.03 ± 2.93	30.80 ± 3.18									
	*p*-value*		0.33	< 0.001	< 0.001									
Autonomous autonomy	Intervention		17.93 ± 1.87	23.50 ± 2.93	22.96 ± 2.35	50.74	< 0.001	0.467	19.42	< 0.001	0.251	24.67	< 0.001	0.298
	Control		18.50 ± 2.75	19.46 ± 2.81	19.43 ± 2.75									
	*P*-value*		0.36	< 0.001	< 0.001									
Competency	Intervention		17.10 ± 3.07	20.53 ± 2.67	19.90 ± 1.80	27.4	< 0.001	0.321	4.79	0.033	0.076	21	< 0.001	0.266
	Control		17.66 ± 2.92	17.73 ± 2.74	18.16 ± 2.30									
	*P*-value*		0.47	< 0.001	0.002									
Relatedness	Intervention		21.26 ± 2.98	22.83 ± 1.08	21.93 ± 2.44	3.25	0.053	0.053	1.89	0.174	0.032	3.2	0.055	0.052
	Control		21.13 ± 3.11	21.13 ± 2.90	21.06 ± 2.71									
	*P*-value*		0.87	0.032	0.2									
Nutrition behavior	Intervention		1.10 ± 0.95	2.30 ± 0.65	2.03 ± 0.61	21.21	< 0.001	0.268	5.71	0.02	0.09	8.29	0.001	0.125
	Control		1.26 ± 0.94	1.56 ± 0.85	1.43 ± 0.85									
	*P*-value**		0.83	0.009	0.031									
Physical activity (moderate level)^a^	Intervention		492 ± 286.40	781.33 ± 249.24	508.66 ± 305.79	4.85	0.009	0.077	9.42	0.003	0.14	10.26	< 0.001	0.15
	Control		406.66 ± 306.81	397.33 ± 296.72	514.66 ± 226.45									
	*P*-value*		0.27	< 0.001	0.93									
Physical activity (low level)^a^	Intervention		310.20 ± 254.67	467.50 ± 191.26	427.90 ± 220.51	7.15	0.001	0.11	8.81	0.004	0.132	1.36	0.259	0.023
	Control		256.30 ± 238.86	370.70 ± 199.26	256.30 ± 163.44									
	*P*-value*		0.4	0.06	0.001									
Sitting^b^	Intervention		8.16 ± 1.31	7.03 ± 1.12	6.70 ± 1.20	8.1	< 0.001	0.123	16.02	< 0.001	0.216	7.35	< 0.001	0.112
	Control		8.23 ± 1.27	8.20 ± 1.29	8.20 ± 1.21									
	*P*-value*		0.11	< 0.001	0.09									
Physical activity (total score)^a^	Intervention		802.20 ± 324.32	1248.83 ± 305.47	936.56 ± 445.94	13.05	< 0.001	0.184	16.48	< 0.001	0.221	6.16	0.004	0.096
	Control		662.96 ± 342.01	768.03 ± 346.88	770.96 ± 302.14									
	*P*-value*		0.11	0	0.1									
Perceived stress	Intervention		25.70 ± 5.25	18.83 ± 3.08	17.90 ± 1.86	55.23	< 0.001	0.488	13.77	< 0.001	0.192	26.65	< 0.001	0.315
	Control		24.60 ± 3.86	23.10 ± 3.57	23.36 ± 3.64									
	*P*-value*		0.36	< 0.001	< 0.001									
Systolic blood pressure	Intervention		149.33 ± 7.84	142.50 ± 8.88	143.16 ± 8.95	6.48	0.003	0.1	0.66	0.419	0.011	1.75	0.181	0.029
	Control		147.67 ± 8.78	144.33 ± 8.58	146.83 ± 10.04									
	*P*-value*		0.44	0.42	0.14									
Diastolic blood pressure	Intervention		96.17 ± 5.03	91.83 ± 7.36	92.33 ± 6.91	4.53	0.014	0.073	0.144	0.706	0.002	1.04	0.353	0.018
	Control		94.83 ± 6.22	92.83 ± 6.90	94.00 ± 7.23									
	*P*-value*		0.37	0.59	0.36									

^a MET-minutes per week. b Hours per day.^

**Figure 1:**
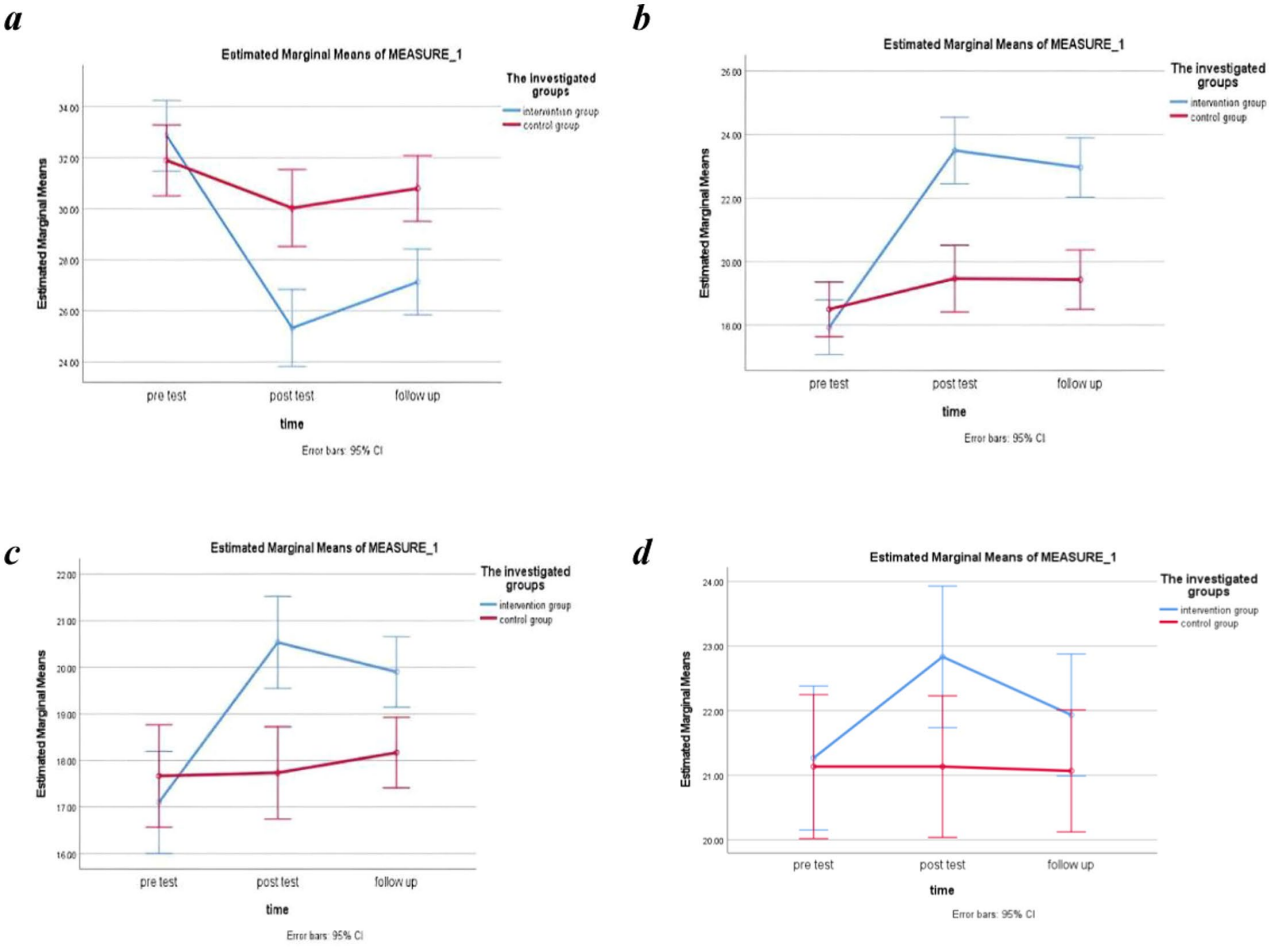
Changes in controlled autonomy **(a)**, autonomous autonomy **(b)**, competency **(c)**, and relatedness **(d)**, before, after, and 2 months after the intervention in the study groups.

The results of the intervention program, based on the theory of self-determination and using the ANOVA test, showed that the effect of the group (between-group effects) on improving controlled autonomy (*F* = 40.70, *p*-value <0.001, η2 = 0.412), autonomous autonomy (*F* = 50.70, *p*-value <0.001, η2 = 0.467), and competency (*F* = 27.40, *p*-value <0.001, η2 = 0.321) was statistically significant (Table 1). The intervention also had a statistically significant effect on time, irrespective of the group effect (within-group effects), for all above-mentioned variables of the self-determination theory, including controlled autonomy (*F* = 10.36, *p*-value = 0.002, η2 = 0.152), autonomous autonomy (*F* = 19.42, *p*-value <0.001, η2 = 0.251), and competency (*F* = 4.79, *p*-value = 0.033, η2 = 0.076) (Table 1). The interaction effect of time and group on improving controlled autonomy (*F* = 15.70, *p*-value <0.001, η2 = 0.213), autonomous autonomy (*F* = 24.67, *p*-value <0.001, η2 = 0.298), and competency (*F* = 21.00, *p*-value <0.001, η2 = 0.266) was also found to be statistically significant (Table 2). The ANOVA test indicated that the effect of the group (between-group effects) resulted in a marginally non-significant improvement in relatedness (*F* = 3.25, *p*-value =0.053, η2 = 0.053). Accordingly, the within-group analysis did not show a significant change (*F* = 1.89, *p-*value >0.05, η2 = 0.032). Additionally, the ANOVA revealed that the effect of time and group interaction on the relatedness variable was marginally non-significant (*F* = 3.20, *p*-value =0.055, η2 = 0.052). Figures 1a–d depict the changes of the constructs of the self-determination theory over time according to the intervention and control groups.

**Figure 2:**
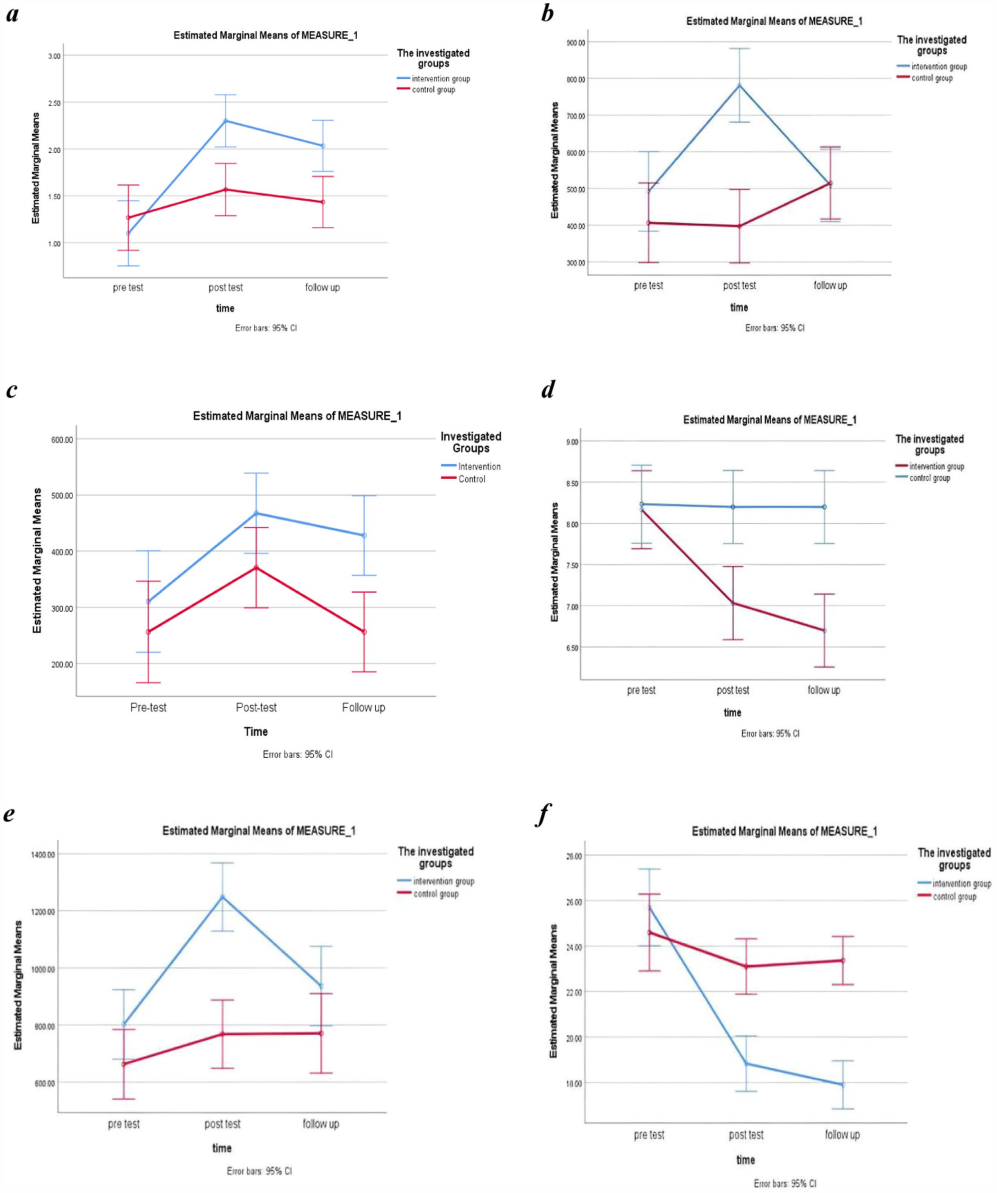
Changes in nutrition behavior **(a)**, moderate level of physical activity **(b)**, low level of physical activity **(c)**, sitting behavior **(d)**, total score of physical activity **(e)**, and perceived stress **(f)** before, after, and 2 months after the intervention in the study groups.

The results of the IPAQ questionnaire revealed that none of the pregnant women engaged in severe physical activity. ANOVA analysis indicated that the effect of group, representing the between-group analysis regardless of time, on lifestyle variables including nutrition behavior (*F* = 2.21, *p*-value <0.001, η2 = 0.27) moderate physical activity (*F* = 4.85, *p*-value =0.009, η2 = 0.078), low physical activity (*F* = 7.15, *p*-value <0.001, η2 = 0.11), sitting (*F* = 8.10, *p*-value <0.001, η2 = 0.123), total physical activity (*F* = 13.05, *p-*value <0.001, η2 = 0.184), and perceived stress (*F* = 5.23, *p*-value <0.001, η2 = 0.49), was statistically significant. Accordingly, the effect of time, i.e., within-group analysis, had a significant impact on nutrition behavior (*F* = 5.71, *p*-value = 0.02, η2 = 0.09) moderate physical activity (*F* = 9.42, *p*-value =0.003, η2 = 0.14), low physical activity (*F* = 8.81, *p*-value =0.004, η2 = 0.13), sitting (*F* = 16.02, *p*-value <0.001, η2 = 0.21), total physical activity (*F* = 16.48, *p*-value <0.001, η2 = 0.221), and perceived stress (*F* = 13.77, *p*-value <0.001, η2 = 0.19). Additionally, the group*time interaction was found to be statistically significant for all lifestyle variables, i.e., nutrition behavior (*F* = 8.29, *p*- value <0.001, η2 = 0.13), moderate physical activity (*F* = 10.26, *p*-value <0.001, η2 = 0.15), sitting (*F* = 7.35, *p*-value <0.001, η2 = 0.11), total physical activity (*F* = 6.16, *p*-value =0.004, η2 = 0.096), and perceived stress (*F* = 26.65, *p*-value <0.001, η2 = 0.32), except for low physical activity (*F* = 1.36, *p*-value =0.26, η2 = 0.02). Figures 2a–f illustrates the trend of changes in lifestyle variables over time based on the intervention and control groups.

**Figure 3:**
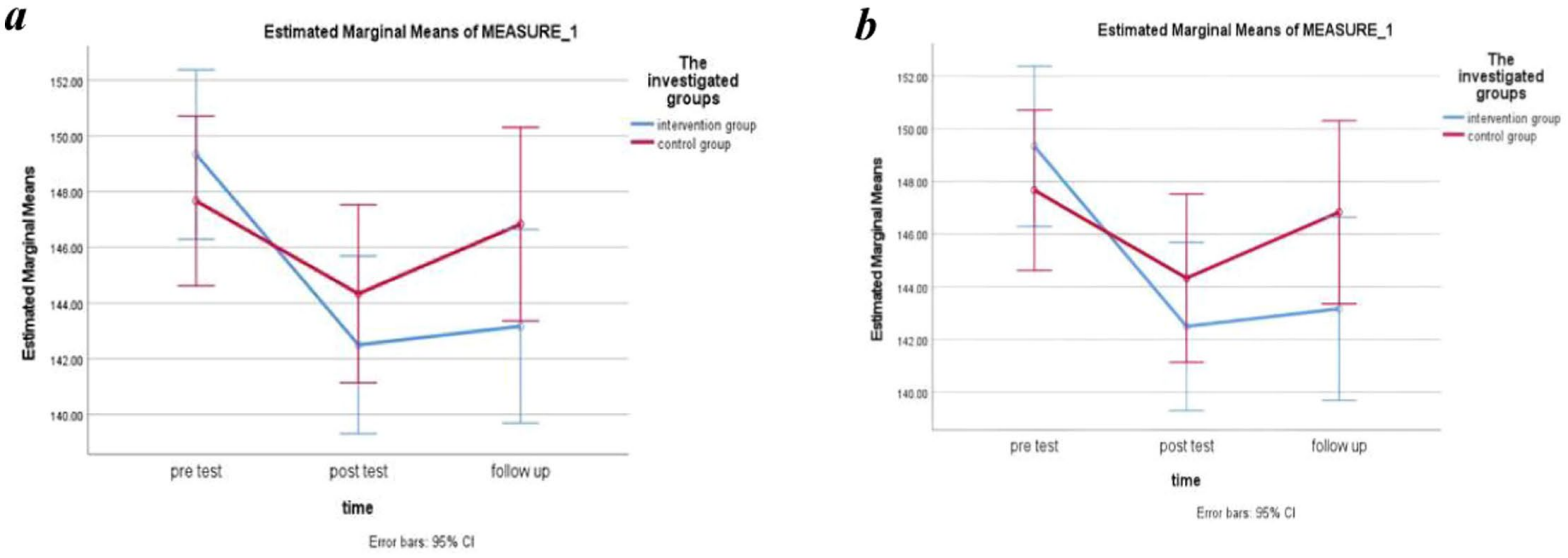
Changes in systolic **(a)** and diastolic **(b)** blood pressure before, after, and 2 months after the intervention in the study groups.

The ANOVA analysis revealed a statistical significant difference between groups for both systolic (*F* = 6.48, *p*- value =0.003, η2 = 0.10) and diastolic blood pressure (*F* = 4.53, *p*- value = 0.014, η2 = 0.073), regardless of time. However, neither the within-group comparison nor the interaction between group and time was significant for either of these two variables (*p-*value >0.05) (Table 2; Figures 3a,b).

## Discussion

This study indicated the favorable effect of the components of self-determination theory (needs for autonomy and competence) using M-Health on improving lifestyle components (diet, physical activity, and stress control) in pregnant mothers. Although the intervention did not have a significant effect on the interaction of time in the group regarding the need for relatedness, which was likely due to the women’s lack of use of strategies related to this section in M-Health, the intervention had a significant effect on the intervention group compared to the control group immediately after the intervention. Additionally, the reduction in systolic and diastolic blood pressure was not statistically significant, which was probably due to the limited duration of the intervention. However, the observed reduction in systolic blood pressure may be clinically meaningful. Maintaining a lower systolic blood pressure during early pregnancy can help reduce the risk of developing early-onset superimposed preeclampsia in women who have chronic hypertension.

### The effect of the intervention on improving autonomy

Autonomy is a fundamental yet contentious concept among basic psychological needs. The need for autonomy means an inherent desire for individuals to experience inner agency in determining what to do, why, how, and when, such that they feel they are the cause of their behavior and possess the right to choose. This includes self-initiation in the initiation, continuation, and management of activities (36).

MansurNejad et al. (37) conducted a study demonstrating that teaching self-determination skills—through goal setting, strategies for achievement, and clarifying the benefits of choices—positively influenced students’ basic psychological needs, particularly enhancing their autonomy, in line with self-determination theory. Thaha et al. (38) utilized self-determination theory strategies in their study, which included framing change as optional, emphasizing seminar attendance, providing choices for behavior change, supporting participant decisions, encouraging lifestyle alignment, and offering positive feedback. They concluded that these educational interventions enhance individuals’ autonomy and boost intrinsic motivation to improve health quality, aligning with the present study (38).

### The effect of the intervention on improving competence

Competence involves effective social interactions and fulfilling responsibilities in novel situations. It includes skills, abilities, and mastery necessary to achieve life goals and attain social acceptance (39).

Zeng et al. (40) assessed a family-based physical activity intervention guided by self-determination theory. The results indicated that facilitators, children, and parents believed that the theory-based intervention successfully promoted co-physical activity and satisfied families’ basic psychological needs during leisure time (40).

Ghasemipour et al. also conducted a study aimed at evaluating the effectiveness of motivational interviewing on satisfying basic psychological needs in heart patients who underwent coronary artery bypass surgery. Their findings indicated that motivational interviewing effectively addressed the needs of autonomy and relatedness, but had no significant impact on the need for competence, which contrasts with the present study. Ghasemipour et al. noted that the depression among coronary artery patients inhibits their ability to satisfy the need for competence, suggesting that treating depression may enhance their sense of competence in managing cardiovascular risk factors (41).

### The effect of the intervention on improving relatedness

Effective relatedness requires two components: valuing the comfort of individuals and their interest in others. Relatedness that lacks care, affection, acceptance, and appreciation does not fulfill the need for relatedness (42).

Mazloumi Mahmood Abad et al. (43) found that their study on motivational interviewing based on self-determination theory did not result in significant differences in relatedness scores or changes over time between intervention and control groups among women of reproductive age, aligning with the present study (43).

According to mothers’ feedback regarding the educational software, one potential reason for this outcome may be the insufficient application of strategies targeting relatedness within the software (such as messaging and challenging peers), This could stem from the uncommon use of educational software for addressing relatedness in health issues, coupled with mothers’ mistrust of medical advice from sources other than their own doctors during pregnancy, due to concerns about not being remembered or receiving inappropriate prescriptions based on their conditions.

To address these issues, it is recommended to increase the number of doctors available to respond to mothers’ inquiries, which would enhance accessibility and trust in the educational software. Additionally, incorporating video communication alongside text options and organizing multiple meetings with the target group could reassure mothers about privacy, fostering trust and encouraging the use of the software, though these approaches require time and further interventions.

Nevertheless, Farmanbar et al. (44) conducted a study to determine the effect of an intervention integrating the Stages of Change model with the Self-Determination Theory on the promoting and maintaining exercise behavior among students. Their findings showed a positive impact of the educational intervention on improving relatedness scores, which does not align with the results of the present study’s result. This discrepancy may be attributed to the integration of the Stages of Change model with the Self-Determination Theory in the intervention. The relationship between these two theories has been documented, indicating that Self-Determination Theory emphasizes processes that align with the Stages of Change model, thereby enhancing individuals’ sense of autonomy, competence, and relatedness, ultimately increasing self-determination during these stages (44).

### The effect of the intervention on promoting a healthy lifestyle

A healthy lifestyle is crucial for reducing health issues and enhancing the quality of life for pregnant women (45). Lifestyle interventions, including healthy dietary habits, physical activity, stress management, and effective healthcare utilization, are particularly recommended for obese pregnant women (46).

#### Diet

Bozorgi et al. (47) implemented a mobile health application that provided educational support for treatment adherence, the DASH diet, and monitoring of blood pressure and physical activity, resulting in improved adherence and blood pressure management among participants (47). While this study aligns with our findings in terms of utilizing software to promote dietary adherence and physical activity, it focused on patients with primary hypertension rather than pregnant women with hypertension. In contrast, Coumans et al. (48) conducted a web-based study utilizing self-determination theory, which found that educational interventions were generally ineffective in improving dietary and physical activity behaviors, potentially due to their limited focus on specific food outcomes (48). This inconsistency with our results highlights the importance of tailored interventions that consider the unique needs of pregnant women with hypertension.

#### Physical activity

In the present study, the M-Health intervention initially led to improvements in moderate-level physical activity among mothers with hypertension, but did not show a significant difference in low-level physical activity immediately after the intervention; however, this changed during the follow-up period as low-level activity increased, likely due to the approaching delivery (24).

Kim et al. (49) similarly showed the effectiveness of a mobile-based self-monitoring program in improving health behaviors and physical activity, although their intervention lasted 6 months compared to 6 weeks in our study (49). Additionally, Fenton et al. (50) found that physical activity counseling sessions promoting autonomy significantly increased motivation and activity levels in patients with rheumatoid arthritis, supporting the notion that motivation is an important component for behavior change (50). While their approach differed from our use of educational software, their findings align with the positive outcomes observed in our study.

#### Stress management

While moderate stress can enhance performance, high levels of stress hinder adaptability and contribute to various health issues, including cardiovascular diseases and weakened immunity (51). Research by Rahimi et al. (52) demonstrated that relaxation training significantly reduced anxiety in high-risk pregnant women through techniques like progressive muscular relaxation and conscious breathing. Similarly, Valiani et al. (53) found that the Body Relaxation Technique (BRT) effectively lowered systolic blood pressure and stress in high-risk pregnant women. This underscores the need for stress management strategies, such as relaxation methods, within mobile application-based health programs for pregnant women.

### The effect of the intervention on lowering blood pressure

Hypertension is a significant chronic disease with high treatment costs in both developing and developed countries. Management requires long-term care plans (54). In this study, the educational intervention reduced systolic and diastolic blood pressure in mothers, but the reduction was not statistically significant, likely due to the short duration of the intervention. Weerahandi et al. utilized DASH Mobile software to monitor diet, blood pressure, weight, and physical activity (55). Their study found no significant change in blood pressure, aligning with the current study’s findings. Conversely, Gong et al.(2020) evaluated mHealth programs aimed at blood pressure control (56). Their intervention included features like medication reminders and alerts for abnormal blood pressure, resulting in a positive impact on reducing blood pressure. This discrepancy may be attributed to their larger sample size of 480 participants and a longer intervention duration of 6 months. Overall, the effectiveness of educational interventions on blood pressure management appears to depend on factors such as duration and sample size (57).

The study’s strengths include its robust study design, detailed intervention components, and appropriate data analysis using repeated measures ANOVA. However, the relatively short intervention duration, modest sample size, lack of long-term follow-up, and some pose limitations. To address these limitations, the authors recommend conducting a larger-scale, longer-duration study to evaluate the sustained impact on blood pressure control and maternal-fetal health outcomes. Although we tried to inform the control group that they should refrain from sharing their information obtained from M-health, the risk of potential information contamination may arise, potentially leading to an underestimation of the effects of our intervention. Exploring the feasibility of integrating the M-Health intervention into routine prenatal care, investigating the specific mechanisms of change, assessing user experience, expanding to diverse populations, and performing a cost-effectiveness analysis are also suggested. These recommendations aim to enhance the intervention’s effectiveness, acceptability, and potential for implementation within the healthcare system, ultimately improving the well-being of hypertensive pregnant women.

## Conclusion

This study presents effectiveness of intervention grounded in self-determination theory to promote healthy lifestyles in hypertensive pregnant women. It can be concluded that M-Health based interventions, which address the basic psychological needs of motivation and the constructs of self-determination theory, including competence, autonomy, and to some extent the need for relatedness, in pregnant women with hypertension, can improve lifestyle factors. In countries like Iran, where pregnant women have widely gained access to smartphones in the last decade, M-Health could be utilized for personalized, consistent, and sustainable primary and secondary prevention interventions to improve prenatal care and benefit maternal and fetal health outcomes.

## Data Availability

The original contributions presented in the study are included in the article/supplementary material, further inquiries can be directed to the corresponding author/s.

## Footnotes

^1^ https://random-allocation-software.software.informer.com/2.0/
